# Spectral responses of gravel beaches to tidal signals

**DOI:** 10.1038/srep40770

**Published:** 2017-01-13

**Authors:** Xiaolong Geng, Michel C. Boufadel

**Affiliations:** 1 Center for Natural Resources Development and Protection, Department of Civil and Environmental Engineering, New Jersey Institute of Technology, Newark, NJ 07102, United States

## Abstract

Tides have been recognized as a major driving forcing affecting coastal aquifer system, and deterministic modeling has been very effective in elucidating mechanisms caused by tides. However, such modeling does not lend itself to capture embedded information in the signal, and rather focuses on the primary processes. Here, using yearlong data sets measured at beaches in Alaska Prince William Sound, we performed spectral and correlation analyses to identify temporal behavior of pore-water pressure, temperature and salinity. We found that the response of the beach system was characterized by fluctuations of embedded diurnal, semidiurnal, terdiurnal and quarterdiurnal tidal components. Hydrodynamic dispersion of salinity and temperature, and the thermal conductivity greatly affected pore water signals. Spectral analyses revealed a faster dissipation of the semi-diurnal component with respect to the diurnal components. Correlation functions showed that salinity had a relatively short memory of the tidal signal when inland freshwater recharge was large. In contrast, the signature of the tidal signal on pore-water temperature persisted for longer times, up to a week. We also found that heterogeneity greatly affected beach response. The response varied from a simple linear mapping in the frequency domain to complete modulation and masking of the input frequencies.

Subsurface biogeochemical processes in coastal aquifers are highly dependent on tidal dynamics, as it has been recognized as a strong driving force for groundwater-seawater recirculation and mixing in beach intertidal zone^1^,^2^,^3^,^4^. Tidal dynamics also generates two distinct saline plumes in the subsurface: the classical saltwater wedge^5^,^6^ and an upper saline plume that overlays a “tube”, whereby fresh groundwater discharges near the low tide mark^7^,^8^,^9^. The presence of waves^10^,^11^,^12^,^13^,^14^ and evaporation^15^,^16^,^17^,^18^ adds an additional level of complexity pore water dynamics^19^,^20^,^21^,^22^,^23^.

Deterministic approaches have been very successful in elucidating physical processes^24^,^25^,^26^,^27^,^28^,^29^. However, they do not readily reveal temporal connectivity, especially on large time scales (e.g., months), which could be achieved through time series analysis (e.g., correlation and spectral analysis) as conducted herein. The approach extracts the essential information from long-term datasets, which could determine which hydrological processes are the major influences in aquifers^30^,^31^,^32^. The approach has provided valuable insights on the hydrology of inland aquifers^33^,^34^,^35^,^36^, and that of coastal aquifers (e.g., groundwater table, salinity and temperature) in the presence of the tidal signals^37^,^38^. Time series analysis using the spectrum and the correlation function has been found to provide valuable spatio-temporal information for tidally influenced beaches; the previous studies revealed that the groundwater table had the signature of the tide ranging from short periods of 12.5 h and 24.7 h to a long period of 14.37 days^39^; the resolution of discrete frequency used in the study was 0.66138 × 10^−2^ cph. The long-term effects of tides on pore-water salinity and temperature in coastal beaches have rarely been monitored and reported. Through the time-series analysis, Tularam and Keeler^40^ found a close relationship between tidal behavior, groundwater depth and salinity levels for the Brisbane River estuary system, Australia. However, in their study, the spectral and correlation analysis was only for a 20-day period, and therefore the long-term effects of tides on pore-water salinity could not be revealed at sufficient details, and will be addressed herein.

We presented yearlong data sets of pore-water pressure, salinity and temperature measured in two gravel beaches in Prince William Sound (PWS), Alaska. Spectral and correlation analyses were performed to provide information identifying fluctuation characteristics of groundwater table, salinity and temperature in tidally influenced beaches. Particular focus was placed on assessing spatial and temporal variability, dominant periods, time lags and memory. These beaches were polluted with the Exxon Valdez oil spill^41^,^42^. Therefore, time series analysis of subsurface hydrodynamics would also be helpful for understanding the long-term oil persistence in these beaches.

## Field measurements

**Figure 1:**
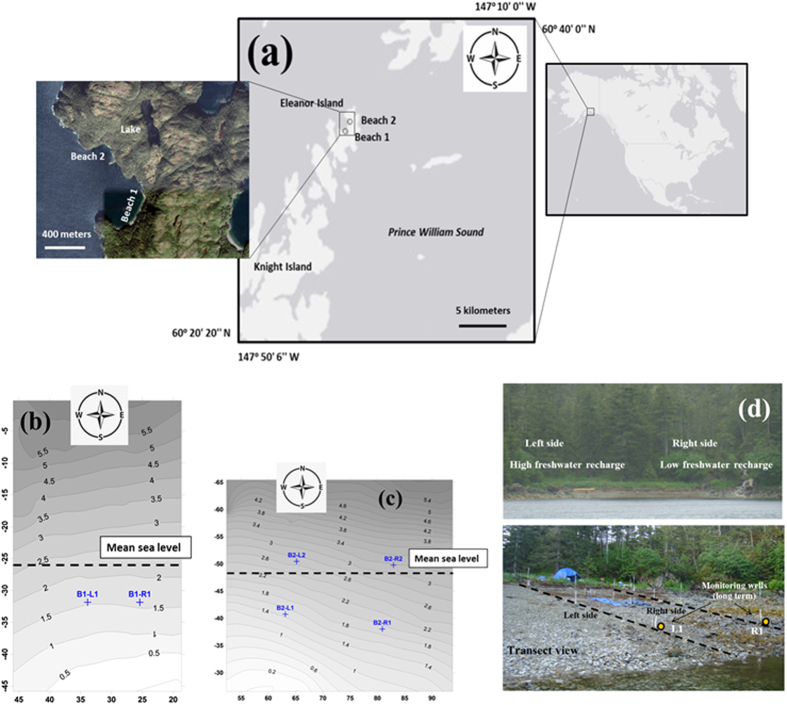
(**a**) Map of two studied beaches. The map was created with ArcGIS, 10.3, (http://desktop.arcgis.com/en/arcmap/). Topographic contours of (**b**) Beach 1 and (**c**) Beach 2, and locations of the observation wells, respectively. (**d**) Photograph of Beach 1 showing the two transects and experimental setup. The photos were taken in the field by the staff (Dr. Xiaolong Geng) in the Center for Natural Resource Development and Protection (CNRDP), New Jersey Institute of Technology (NJIT). The lowest low tide mark during measurement was assigned as the elevation datum (0.0 m). The contours were created with Surfer 8.0 software. All dimensions are in metres.

The two beaches investigated were located in Prince William Sound (PWS), Eleanor Island (Fig. 1). The surface materials of the beaches (i.e., top 30–60 cm depth depending on location) were composed of sand, gravel, pebble and cobble varying from 0.25 to 300 mm. These were intertwined between 60 to 120 cm boulders, which were typically spaced by a few meters. The percentage of large sediments and boulders increased going seaward. The average slope of the intertidal zone of each beach was 10% (Figure S1). The maximum tidal range was approximately 4.7 m. Pits were hand-dug down to a depth of approximately 0.6 m whenever possible, and then a perforated PVC pipe containing the sensor was placed into the beach. In Beach 1, two pits were dug (Fig. 1), and a CTD-Diver sensors (data Logger-DI271, Schlumberger) was used within the PVC pipe to monitor the pressure head, temperature, and salinity of pore water. In Beach 2, four pits were dug (Fig. 1), and a Mini-Diver sensor (data Logger-DI501, Schlumberger) was placed into the perforated PVC pipe to monitor the pressure head and temperature of pore water. Measurements were conducted from August 2007 through June 2008 for both beaches. The hydrodynamics of these beaches was investigated in our previous work^42^,^43^,^44^,^45^.

## Results and Discussions

**Figure 2:**
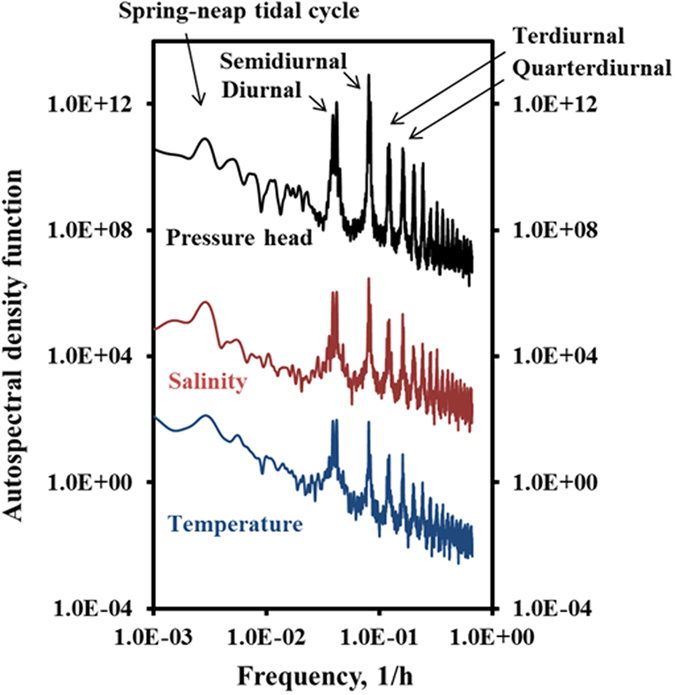
The auto-power spectra of pore-water pressure (m^2^·h), temperature (°C·h), and salinity (g^2^·h/L^2^) as a function of frequency in B1-L1, located on the left side of Beach 1. Pressure head and salinity data were multiplied by 10^10^ and 10^2^, respectively, to separate the spectral density curves in the same Figure. The spectra analyses were performed in MATLAB after detrending the time series.

Analysis of auto-/cross- spectral and correlation was performed by Fast Fourier Transform (FFT) using MATLAB. Figure 2 shows auto-power spectra of the pressure head, temperature and salinity at sensor B1-L1 (those of B1-R1 and Beach 2 were essentially the same, see Figure S2). Five distinctive peaks were observed at frequencies of 0.0028, 0.04, 0.082, 0.12 and 0.16 h^−1^, which correspond to the spring-neap tidal cycle (14.8 d), the diurnal (24.8 h), semidiurnal (12.4 h), ter-diurnal (8.2 h) and quarter-diurnal (6.1 h) periods, respectively. For frequency higher than quarter-diurnal, many peaks were also observed but their amplitudes were relatively smaller in comparison to the former, which indicates that most of the energy (i.e., information) of the system was contained at periods larger than quarter diurnal.

**Figure 3:**
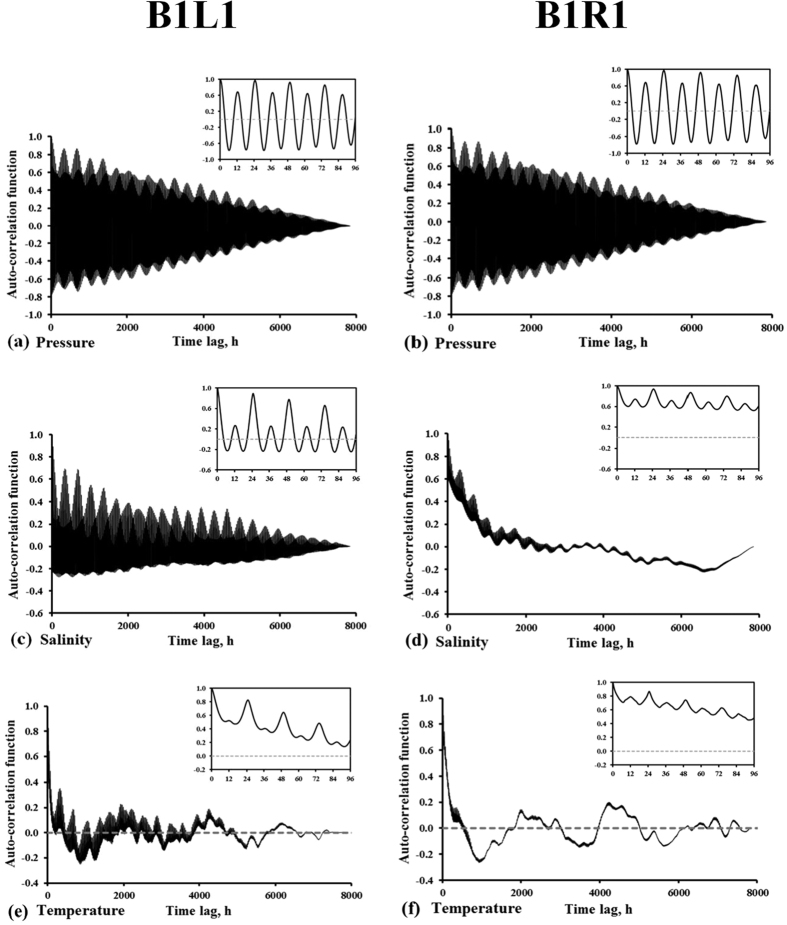
Auto-correlation of (**a**) and (**b**) pore-water pressure, (**c**) and (**d**) salinity, and (**e**) and (**f**) temperature as function of time, at the two sensors B1L1 and B1R1. The first 96 hours of lags are shown in the insets.

Auto-correlation functions of pressure head, salinity and temperature at B1-L1 and B1-R1 are presented in Fig. 3. A strong periodicity manifested at 24.8 hours at all sensors, capturing therefore the diurnal tidal. Semi-diurnal correlation (period of 12 hours) was also noted for salinity, especially at B1-L1 (Fig. 3c), but it was more diffuse for temperature. Although the beach was subjected to semi-diurnal tides, the periodic variations of pressure head, temperature and salinity seemed to be dominated by diurnal tidal actions, which was probably due to daily inequity of semidiurnal tides where successive tides had different amplitudes. The daily unequal tidal amplitudes made the self-similar behavior of pressure head, temperature and salinity more apparent at diurnal frequency rather than that of semidiurnal. These phenomena to some extent reflected important role of amplitude of tides in altering transport behavior of subsurface solute and heat in the beach.

The autocorrelation function for pressure was essentially the same for sensors B1-L1 and B1-R1. The autocorrelation function for salinity at B1-L1 (Fig. 3c) exhibited strong variation with the time lag, from 0.2 to almost 1.0 (at the diurnal period), but that of B1-R1 remained between 0.6 and 1.0. Semi-diurnal peaks were present for the salinity at both B1-L1 and B1-R1. The strong periodicity at B1-L1 in comparison to B1-R1 is due to the presence of freshwater groundwater inflow into the beach at the left transect in comparison with the right transect^42^,^46^. The autocorrelation function of salinity at B1-R1 decreased slowly with time suggesting a weak coupling between tidal forcing and pore water salinity at that location. In coastal beaches, intertidal salt structure was significantly altered by tides. At high tide, seawater infiltrated into the beach, mixed with the brackish groundwater, and generated upper saline plume in subsurface. During the low tide, the ‘signature’ of tides on pore-water salinity would be partially removed from the beach as the mixture of seawater-groundwater in the intertidal zone would be diluted by the freshwater flowing from inland, and gradually discharged into the sea. With larger inland freshwater recharge, the pore-water salinity due to high tides would decrease rapidly from the beach during low tides. This flushing was reflected by larger decrease in autocorrelation during the low tide (Fig. 3c). Therefore, our results indicate that salinity had a relatively short memory of the tidal signal when inland freshwater recharge was large and relatively long memory when the inland freshwater recharge was small.

The autocorrelation function of temperature decreased rapidly with the time lag for B1-L1 and slower for B1-R1, and both sensors showed strong diurnal effects and diffuse semi-diurnal effects, as could be noted by the small increases at 12 hours, 36 hours, etc. Both salinity and heat were transported by advection and dispersion. The sediments played only a minor role for the transport of salinity, as the sand and gravel grains had very little cation exchange capacity^47^. However, the grains played an important role for heat transport by conduction (i.e., heating and cooling of the grains)^48^. As a result, the temperature variation was slower than that of salinity, as the sediment grains played a thermal buffering role. The thermal buffering due to heat exchange with surrounding sediments has been emphasized in many theoretical and experimental studies^49^,^50^. This is evident in comparing Fig. 3e and f to Fig. 3c and d, respectively, where the autocorrelation function of temperature was smoother than that of salinity for the corresponding sensor. Thermal buffering in a sense played the role of a high-pass filter removing the relatively high frequency fluctuations such as tides.

At Beach 2 (Figures S3 and S4), one notes that the autocorrelation function of temperature at landward sensors (i.e., B2-L1 and B2-R1) crossed the zero faster than at the seaward sensors (i.e., B2-L2 and B2-R2). This is probably due to high advection at the landward sensors. The upper layer, which has a high permeability, became thicker moving landward. Therefore, although the sensors installed at the same depth from the ground surface, the landward ones were located in or closer to the upper layer of the beach (shown in Figure S1). These sensors exhibited clearer response to seawater infiltration at high tides and inland freshwater recharge at low tides. Numerical simulations of this beach, which were reported in Bobo, *et al*.^45^, showed that landward pore-water advection during rising tides was comparable at both locations, but seaward advection during ebbing tides was larger at the landward locations. This behavior was common in beaches at PWS^42^,^44^ and other beaches, and explained why contaminants flushed fast from the upper and mid-intertidal zone and persisted in the lower intertidal zone^51^.

**Figure 4:**
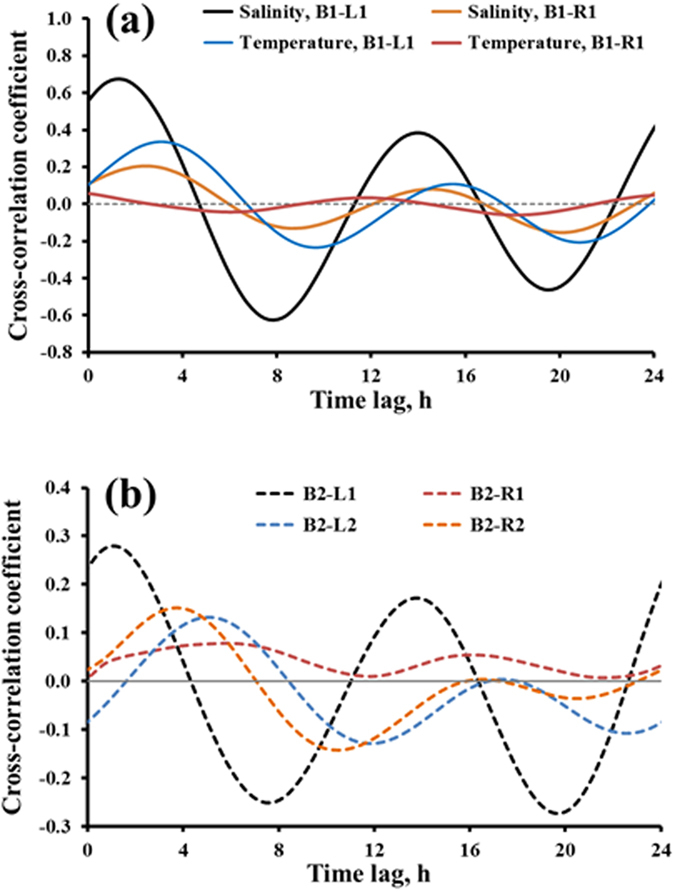
Cross-correlation coefficient function in the first 24 hours using pressure as input (**a**) For B1-L1 and B1-R1, location in Beach 1 with temperature and salinity as output and (**b**) For B2-L1, B2-L2, B2-R1, and B2-R2, located in Beach 2, with only temperature as output (salinity data were not taken at Beach 2). Note that the y-axis scales are different.

Figure 4a shows the cross-correlation functions using pressure head as input and salinity and temperature as output for Beach 1. Only 24 hours of lags is shown for clarity. On the left side of the beach, the maximum cross-correlation coefficients for the output signals of salinity and temperature were 0.7 and 0.35 with a delay time of 1.5 h and 3.0 h. The results indicate that salinity had stronger correlation with pressure head, and faster response to pressure head than temperature had. This is due to the thermal inertia of the sediments, which both dissipated the temperature signal and shifted it in time, as discussed earlier. On the right side of the beach, the cross-correlation coefficient of salinity (B1-R1) had a maximum value around 0.2 with a lag of 2.25 hour. That of temperature at B1-R1 was almost zero throughout, and had a lag of almost 12 hours. Thus, the impact of tide on pore water hydrodynamics was small on the right transect of Beach 1, which might explain the persistence of oil there, as reported by Boufadel, *et al*.^41^.

The impact of beach properties is also observed in Fig. 4b, which reports the cross correlation of pressure-temperature at four locations in Beach 2 (note that no salinity measurement was available at that beach). The largest value was equal to 0.3 at B2-L1, which was comparable to that of pressure-temperature at B1-L1 (Fig. 4a). The maxima at the remaining sensors were around 0.1 suggesting small advection to these sensors. For sensors at the same cross-shore location (B2-L1 and B2-R1; B2-L2 and B2-R2) the cross correlation was larger at left sensors. The cross-correlation function reflected the intensity of the input-output link and its linearity. At the locations with small correlation coefficients (e.g., at B1-R1 and in Beach 2), the tidal signal for salinity and temperature was filtered out during its passage due to poor hydraulic connection there; therefore, the pore-water salinity and temperature at these locations behaved nonlinearly in response to tides. In contrast, at locations with large correlation coefficient (e.g., B1-L1), pore water salinity tended to vary more linearly with the tidal signal. The cross-correlation functions reported herein indicate significant spatial variation of pattern of salinity and temperature change in response to tidal signal. Considering the short distances between these sensors (5 meters across and 20 meters along the shore), one concluded that meter-scale heterogeneity was a major control on these beaches. Based on our earlier investigation, the porosity of the grains decreased sharply with depth resulting in two layers in the beach; a high-permeability surface layer underlain by a low-permeability layer. The depth of the high permeability layer varied between 0.6 m and 1.6 m, which could significantly affect local advection and dispersion of solute in the beach. Therefore, our results suggest that besides long-term monitoring, greater spatial sampling at various depths would be needed to fully understand the dynamics of groundwater flow and solute transport processes in these beaches.

**Figure 5:**
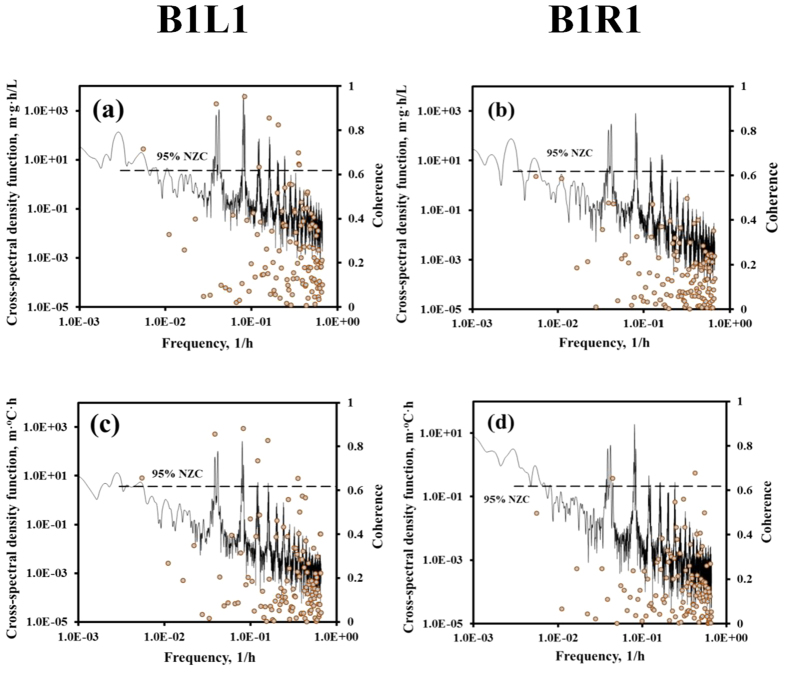
The cross-spectral density (solid lines shown as primary axis) and corresponding coherence (symbols shown as secondary axis) using pressure head as input and salinity (**a** and **b** for B1L1 and B1R1, respectively) and temperature (**c** and **d** for B1L1 and B1R1, respectively) as output. The short dash lines indicate the 95% non-zero coherence level (NZC).

Figure 5 reports the cross-spectra of pressure-salinity and pressure-temperature at Beach 1. Those of Beach 2 are reported in Figure S5. The peaks noted for the auto-spectra (Fig. 2) were evident at frequency of 0.04 and 0.082 h^−1^, corresponding to the diurnal and semi-diurnal tidal periods. The results indicate the periodic response of pore-water salinity and temperature to tidal fluctuations. For pressure-salinity and pressure-temperature at B1-L1, the values of coherence exceeded the 95% non-zero confidence level at the dominant frequencies (of Fig. 2), indicating strong correlation between pore-water pressure and salinity at these frequencies. This suggests more or less a linear mapping from the pressure frequency domain to the salinity frequency domain at B1-L1, that pressure forcing at given frequencies produced a response in salinity and temperature at the same frequency at that location. As advection is simply a translation in the time domain, it would produce an output that resembles the input, but dispersion causes frequency modulation accompanied by a decrease of the spectral magnitude at high wave numbers^52^. Thermal conductivity would tend to buffer major changes, and its behavior can then be approximated by a decay, where one also notes additional frequency shift accompanied by a decrease in the magnitude^52^. It is thus concluded that advection was the dominant transport mechanisms at B1-L1, which is in agreement of the findings of Li and Boufadel^42^. For B1-R1, the coherence for the pressure-salinity (Fig. 5b) and pressure-temperature (Fig. 5d) was less than 95% at the dominant frequencies, suggesting modulation of these frequencies due to dispersion, and that a one-to-one relation between input and output did not exist at B1-R1. For Beach 2 (Figure S5), the coherence for the diurnal component of the pressure-temperature cross-spectral density was >95% at all sensors. However, it was larger than 95% for the semidiurnal components only at the left transect of Beach 2 (B2-L1 and B2-L2). Thus, major frequency modulation at the right side of Beach 2 took place.

## Conclusion

Spectral and correlation analysis was carried out to identify temporal behavior of pore-water pressure, temperature and salinity. Yearlong data sets measured at beaches in Alaska Prince William Sound were used for the time-series analysis. The results of the auto-power spectra revealed that the response of the beach system could be characterized by fluctuations of embedded diurnal, semidiurnal, terdiurnal and quarterdiurnal tidal components.

Correlation functions provided the information that large inland freshwater recharge shortened the memory of the pore-water salinity to the tidal signal, while compared to the salinity, due to the thermal conductivity of sediments, the signature of the tidal signal on the pore-water temperature persisted for longer times, up to a week. Therefore, the response of the pore-water salinity to tidal signal was faster than that of temperature. Our findings indicated that the ecosystem processes in these beaches were well adapted to short-term rapid salinity variation rather than the temperature. The cross-spectra analysis revealed more or less a linear mapping from the frequency domain of pressure to that of salinity at B1-L1. However, at other locations (e.g., B1-R1 and B2), pore-water salinity behaved nonlinearly in response to the tidal signal, which is most likely due to the poor hydraulic connection. These results should not be viewed as reflecting a limitation of the approach, rather an advantage, as our approach allowed us to characterize the local heterogeneity in the beach. By considering only the autocorrelation function (Fig. 3 and Figures S3 and S4), one might be able to stipulate weak dependence between water pressure and either of salinity or temperature. However, only by using the cross-correlation function (Fig. 4) that one can be certain of their correlations. For example from Fig. 3, the pressure and temperature at B1L1 seemed to behave similar to those at B1R1. However, Fig. 4 showed that the cross correlation between pressure and temperature at B1L1 was almost 40% while it was less than 5% at B1R1.

Validation of numerical models by comparison to the observation has been a crucial step in hydrology^9^,^42^,^53^,^54^. However, the findings herein revealed that at some locations, the concentration and/or temperature were only weakly related to pore-water hydraulics. Thus, matching observations at these locations for a short duration (say a week or even a month) might be viewed as matching to only one “realization”. In such a case, there would not be an assurance that calibrated numerical models can capture the long term (or ensemble) behavior. Alternatively, it might be prudent to test whether numerical models are able to reproduce the statistics of the data (spectra and correlations). The simulation of heat transport is of importance due to climate change, and the findings herein suggested that the modeling of heat transport in coastal systems is more challenging than that of solute transport, which is due to thermal buffering capacity of the sediments. In this paper, spectra and correlation function was computed using Fast Fourier Transform (FFT). There are also other methods that could be used such as spectral analysis 2^nd^ order and wavelet analysis^55^,^56^,^57^,^58^, which would need to be considered and compared to the results presented in this paper.

### Data Availability

The data for this paper are available upon e-mail request (nrdpcenter@gmail.com) at Center for Natural Resources Development and Protection.

## Additional Information

**How to cite this article**: Geng, X. and Boufadel, M. C. Spectral responses of gravel beaches to tidal signals. *Sci. Rep.* **7**, 40770; doi: 10.1038/srep40770 (2017).

**Publisher's note:** Springer Nature remains neutral with regard to jurisdictional claims in published maps and institutional affiliations.

## Supplementary Material

Supplementary Information
