# Supplementary material for: Spectral responses of gravel beaches to tidal signals
Source: Sci Rep. 2017 Jan 13;7:40770. doi: 10.1038/srep40770 (PMC5234018; doi:10.1038/srep40770)
Supplement: Supplementary Information [file srep40770-s1.pdf]

Spectral responses of gravel beaches to tidal signals

Xiaolong Geng[1] and Michel C. Boufadel[1*]

1. Center for Natural Resources Development and Protection,
Department of Civil and Environmental Engineering, New Jersey Institute of Technology, Newark, NJ 07102, United States

*: Corresponding author. boufadel@gmail.com, Center for Natural Resources Development and Protection, New Jersey Institute of Technology, Newark, NJ 07102, United States

Supplementary Material

This supplementary provides additional data from the field site at Eleanor, Prince William Sound, Alaska. The material is organized as five figures: one for beach topography (Supplementary Figures 1), and four for the results of spectral and correlation analyses (Supplementary Figures 2-5). Beach transect topography survey is shown in Supplementary Figure 1. Addition results of auto-spectra, auto-correlation, and cross spectra analyzed for Beaches 1 and 2 are shown in Supplementary Figures 2-5.

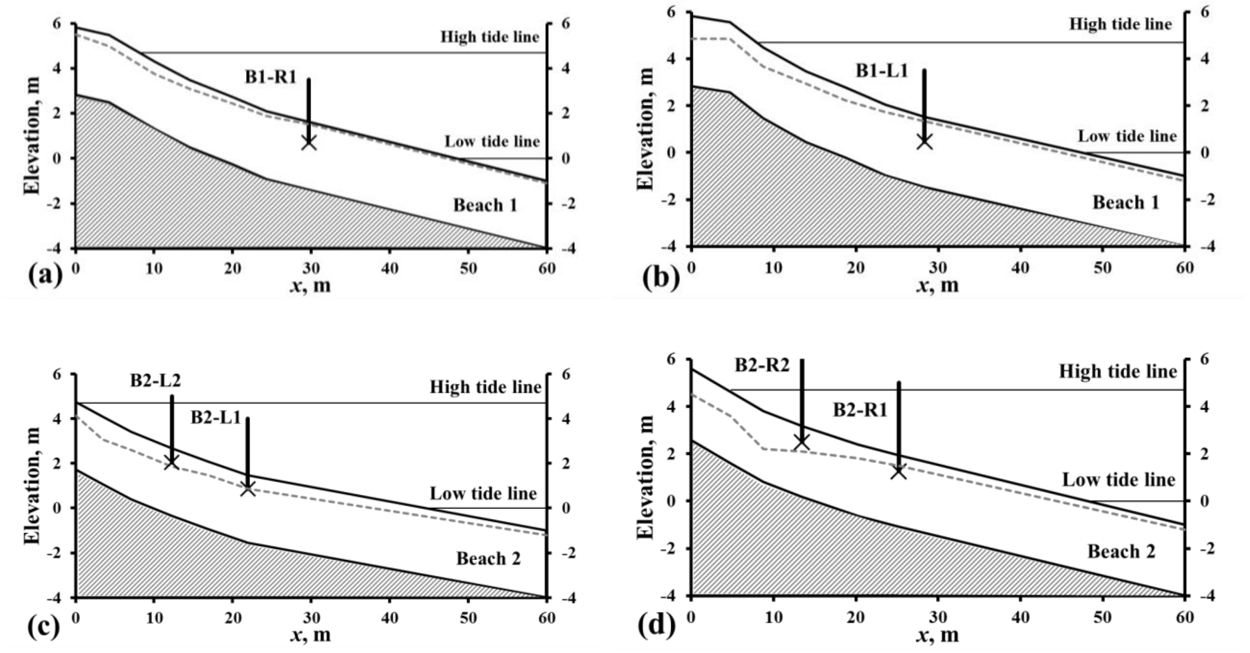

Figure S1: Location of sensors at (a) Right and (b) Left transects at Beach 1, and (c) Right and (d) Left transects located at Beach 2. Field studies (Li and Boufadel, 2010; Bobo et al., 2012) indicate the presence of two layers within the beach: the upper layer consists of loose gravel and coarse sand and the lower layer is compacted silt and fine sands. The interface of the two layers is delineated by dashed lines shown in the Figure. Measurements were conducted from August 2007 through June 2008 for both beaches. Pore-water pressure, temperature and salinity were measured at Beach 1 in two wells, B1-L1 and B1-R1. Pore-water pressure and temperature were measured at Beach 2 from four wells B2-L1, B2-L2, B2-R1, and B2-R2.

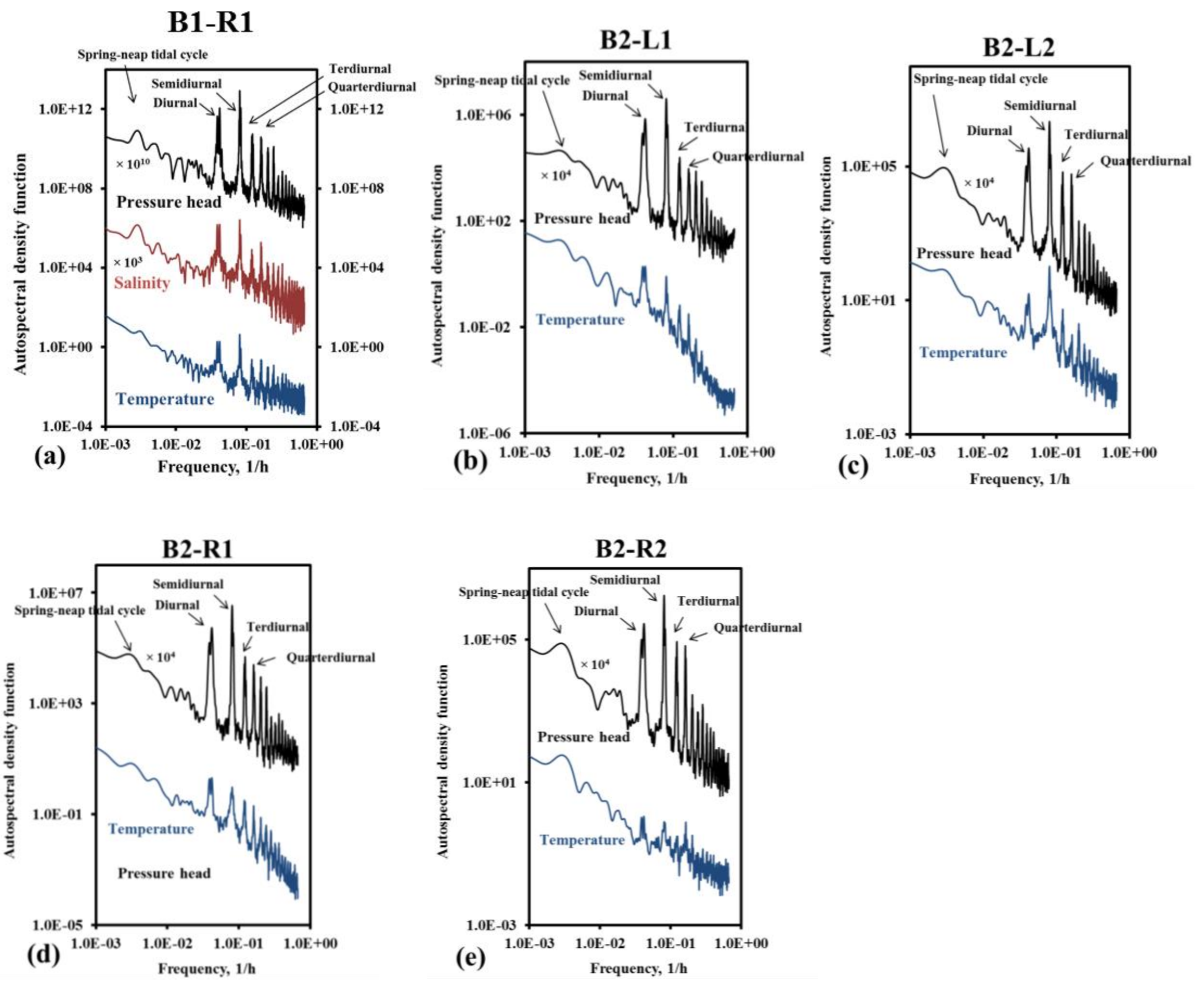

Figure S2: The auto-power spectra of pore-water pressure, temperature, and salinity (if salinity data is available) as a function of frequency in (a) B1-R1, located on the right side of Beach 1, (b-e) B2-R2, B2-R1, B2-L2, B2-L1, located on Beach 2. Temperature and salinity data were multiplied by very large number, in order to separate the spectral density curves in the same Figure.

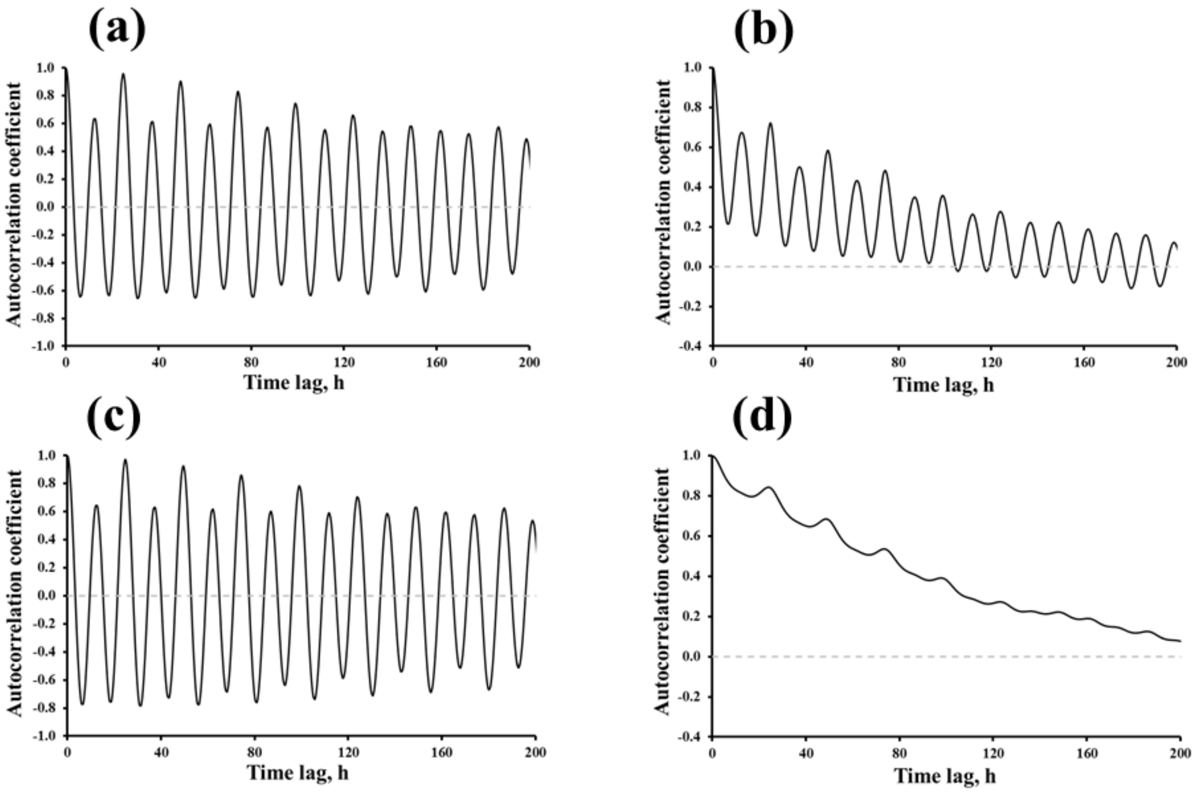

Figure S3: Auto-correlation of (a) and (b) pore-water pressure, and (c) and (d) temperature as a function of time, at the two sensors B2-L1 and B2-L2.

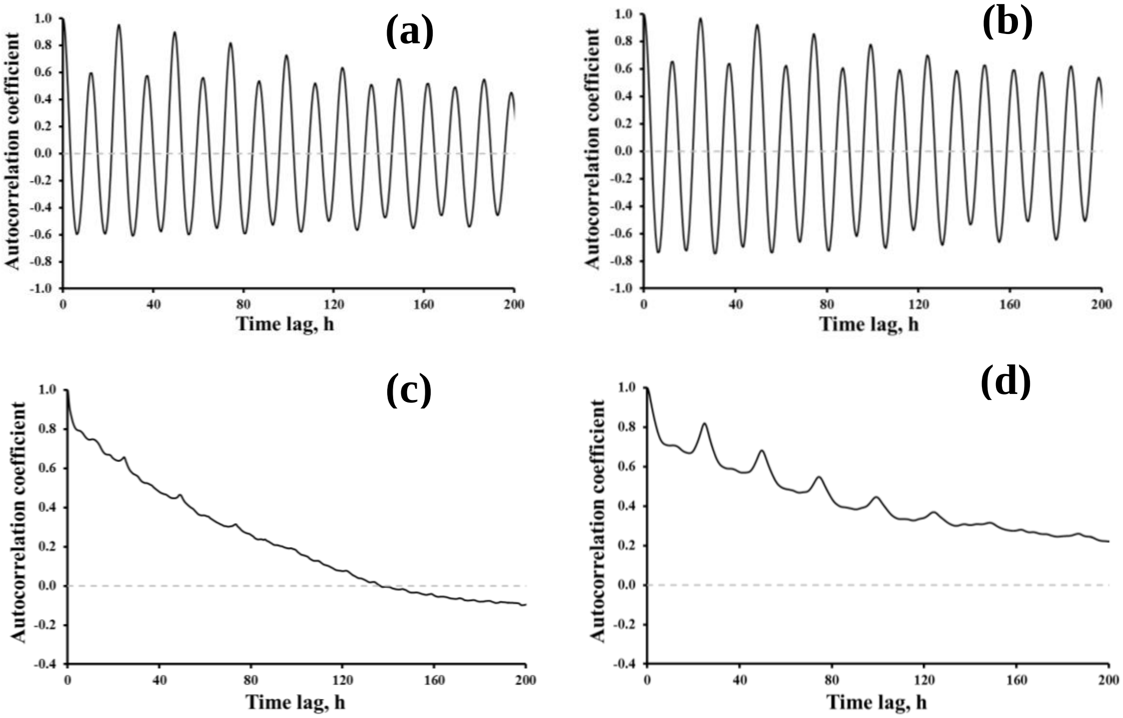

F**igure S4:** Auto-correlation of (a) and (b) pore-water pressure, and (c) and (d) temperature as a function of time, at the two sensors B2-R1 and B2-R2.

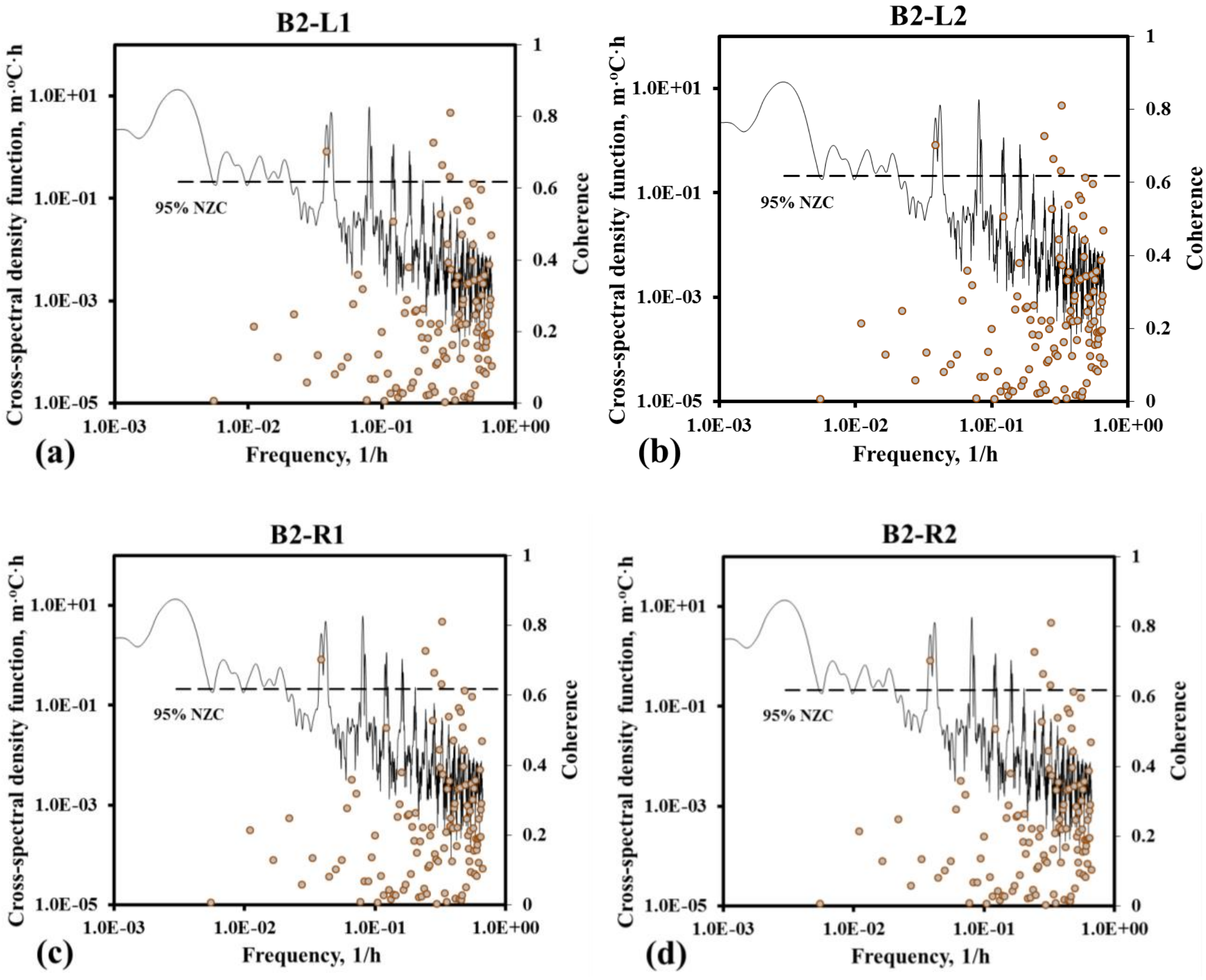

Figure S5: The cross-spectral density (solid lines shown as primary axis) and corresponding coherence (symbols shown as secondary axis) using pressure head as input and temperature as output for (a-d) wells B2-L1, B2-L2, B2-R1, B2-R2, located on Beach 2. The short dash lines indicate the 95% non-zero coherence level (NZC). The spectra analyses were performed in MATLAB.
